# Characterization of *Salmonella enterica* serovar Isangi from South Africa, 2020–2021

**DOI:** 10.1186/s12879-023-08786-9

**Published:** 2023-11-13

**Authors:** Asive Myataza, Juno Thomas, Anthony M. Smith

**Affiliations:** 1 Centre for Enteric Diseases, National Institute for Communicable Diseases, Division of the National Health Laboratory Service, Johannesburg, South Africa; 2 Department of Medical Microbiology, Faculty of Health Sciences, University of Pretoria, Pretoria, South Africa

**Keywords:** *Salmonella enterica*, Serovar Isangi, Isangi, Africa, South Africa, ESBL

## Abstract

**Background:**

We describe the genotypic characteristics and antimicrobial resistance (AMR) determinants of *Salmonella enterica* serovar Isangi (*Salmonella* Isangi) clinical isolates in South Africa from 2020 through 2021.

**Methods:**

During the years 2020 to 2021, the Centre for Enteric Diseases of the National Institute for Communicable Diseases, a national reference centre in South Africa for human infections resulting from enteric bacterial pathogens, investigated a total of 3549 clinical isolates of *Salmonella* species. Whole genome sequencing (WGS) was performed using Illumina NextSeq Technology. WGS data was analyzed using Centre for Genomic Epidemiology-based tools and EnteroBase web-based platform. Genotypic relatedness and cluster analysis was investigated based on core-genome multilocus sequence typing.

**Results:**

Forty-nine isolates were confirmed to be *Salmonella* Isangi, with most submitted from Gauteng Province (24/49, 49%). The most prevalent sequence type was ST335 (48/49, 98%), and the remaining 1 isolate was ST216. All ST335 isolates were genotypically multidrug-resistant (MDR), with resistance to fluoroquinolones, chloramphenicol, trimethoprim-sulfamethoxazole and tetracycline; the ST216 isolate was resistant only to aminoglycosides. All ST335 isolates carried ESBL genes, the most common being *bla*_CTX-M-15_. Five clusters (consisting of isolates related within five allele differences) were detected, all being ST335.

**Conclusions:**

Most *Salmonella* Isangi isolates in South Africa are MDR and ESBL-positive. Ongoing monitoring of the epidemiology and AMR profile of this serovar is important for public health and treatment guidelines.

**Supplementary Information:**

The online version contains supplementary material available at 10.1186/s12879-023-08786-9.

## Background

Non-typhoidal *Salmonella* (NTS) infections present a major public health concern and are linked to foodborne gastroenteritis worldwide. Invasive NTS disease, include septicemia, endovascular infection, and meningitis. These can cause significant morbidity and mortality in adults, children, and immunocompromised individuals, particularly in sub-Saharan Africa [1]. While most *Salmonella* infections are self-limiting, invasive salmonellosis often require antibiotic treatment [2]. Emerging multidrug-resistance (MDR) against commonly used antibiotics, including ampicillin, chloramphenicol, and sulfamethoxazole, has necessitated the use of fluoroquinolones and extended-spectrum cephalosporins (ceftriaxone and cefotaxime) [1]. Consequently, this has promoted the development of extended-spectrum β-lactamases (ESBLs) and class C beta-lactamases (AmpC)-production in *Salmonella enterica* (*S. enterica*) serovars including *Salmonella enterica* serovar Isangi (*Salmonella* Isangi) [3]. ESBLs and AmpC are enzymes that confer resistance against most beta-lactam antibiotics, including penicillins, and cephalosporins, with ESBLs comprising of isolates that exhibit co-resistance to other classes of antibiotics such as aminoglycosides, fluoroquinolones and sulfonamides. ESBLs can be inhibited by β-lactamase inhibitors such as clavulanic acid, while AmpC activity is not affected by ESBL inhibitors [4]. Although ESBL-production is predominantly associated with other species in the *Enterobacteriaceae* family, the emergence of ESBLs in *Salmonella* Isangi has become increasingly more prevalent in South Africa [2]. ESBLs in *Salmonella* Isangi in South Africa (SA) were first reported in 2001 and have since gained interest due to transmissible mobile genetic elements that produce a variety of ESBL enzymes, including *bla*_*TEM*_, *bla*_*SHV*_, *bla*_*CTX-M-15*_ and *bla*_*CTX-M-37*_ [3, 5]. Plasmid-mediated AmpC-type β-lactamases producing *Salmonella* strains have been detected in the USA [6]. Identification of the presence of ESBLs and AmpC β-lactamases in *Salmonella* species is of significant therapeutic importance, particularly in SA where the majority of *Salmonella* Isangi isolations are MDR. The aim of this study was to identify genotypic characteristics, molecular strain relatedness, as well as the genetic basis of antimicrobial resistance determinants of *Salmonella* Isangi clinical isolates collected from SA in year 2020 through 2021.

## Methods

### Study location

The Centre for Enteric Diseases (CED) of the National Institute for Communicable Diseases (NICD) participates in national laboratory-based surveillance for isolates of *Salmonella*, where > 200 diagnostic clinical microbiology laboratories across the country voluntarily submit various enteric bacterial isolates to the CED.

### Biographic distribution of samples

For the current study, clinical *Salmonella* isolates for the years 2020 and 2021 were investigated. The demographic information of the cases including age, sex, and region (province; see supplementary Fig. 1) and sample source were recorded. A South African map outlining the various province the isolate came from is shown in Supplementary Fig. 1. Age was classified into three categories: children (0–17 years), adults (18–59 years) and senior adults (60 years and above).

### Phenotypic and genetic analysis

These isolates were phenotypically identified using the VITEK-2 COMPACT 15 automated microbial identification system (bioMérieux, Marcy-l′Étoile, France). Subsequently, VITEK identified isolates were sub-cultured onto blood agar (Diagnostic Media Products, National Health Laboratory Service, South Africa). Thereafter, genomic DNA of *Salmonella* isolates was extracted using the Invitrogen™ PureLink™ Microbiome DNA Purification Kit (Thermo Fisher Scientific, Waltham, USA). Genomic DNA was then subjected to WGS using Illumina NextSeq (Illumina, San Diego, CA) next generation sequencing technology. WGS data of all genomes was analyzed using various bioinformatics tools available at the Centre for Genomic Epidemiology (https://www.genomicepidemiology.org/) web-based platform. Generated raw reads (FastQ files for paired-end reads) were uploaded and automatically assembled in EnteroBase web-based platform (http://enterobase.warwick.ac.uk/species/index/senterica). We analyzed the presence and distribution of acquired genes associated with antimicrobial resistance to the following classes of antimicrobials: aminoglycosides, β-lactams, fluoroquinolone and aminoglycoside, fosfomycin, macrolide, phenicol, quinolone, rifampicin, sulphonamide, tetracycline, and trimethoprim. Genotypic relatedness and cluster analysis of isolates was investigated using the core-genome multilocus sequence typing (cgMLST) approach using the ‘cgMLST V2 +HierCC V1’ scheme of EnteroBase. The phylogeny and genetic relatedness of isolates was depicted using a GrapeTree-generated minimum spanning tree using the ‘MSTree V2’ algorithm (https://bitbucket.org/enterobase/enterobase-web/wiki/GrapeTree) [7]. A cluster of isolates was defined as ≥3 isolates showing ≤5 allelic differences, as determined following cgMLST analysis and visualized on a GrapeTree-generated minimum spanning tree.

## Results and discussion

### Phenotypic and genotypic prevalence of *Salmonella* Isangi

For the years 2020 to 2021, a total of 4945 clinical cases of *Salmonella* infection were notified to CED, NICD. For these cases, only 3637 viable cultures were received by the CED, of which the cultures were stored in glycerol stock at − 70 °C until ready to use. At the time for whole genome sequencing (WGS), only 3549 cultures could be revived and were analyzed further using WGS. Forty-nine *Salmonella* isolates were identified as *Salmonella* Isangi by analysis of WGS data. Gauteng Province had the highest number of *Salmonella* Isangi isolates (24/49, 49%) with other isolates belonging to the following regions: Limpopo Province (*n* = 8), Mpumalanga Province (*n* = 7), KwaZulu-Natal Province (*n* = 3), North-West Province (*n* = 2), Eastern Cape Province (*n* = 3), and Free State Province (*n* = 2). The sources of the isolates were stool (22 isolates), blood culture (10 isolates), urine (7 cultures), rectal swab (2 isolates), and other (8 isolates). A total of 36 isolates were non-invasive (stool) while 13 isolates were invasive (blood, CSF) as indicated by the metadata of each patient. The age distribution was as follows: children (*n* = 11), adults (*n* = 30) and senior adults (*n* = 8); with a total of 22 females, 23 males and 4 gender unclassified cases.

### Genetic characteristics of isolates

A total of 25 different AMR profiles (AMR profile 1–25) which group AMR gene combinations or resistance trends were identified. WGS revealed that *Salmonella* Isangi isolates (*n* = 48/49, 98%) linked to AMR profile 1 to 24 were MDR. Isolates from these AMR profiles harbored several resistance genes conferring resistance to aminoglycosides (*aac(6′)-Iaa*, *aph(6)-Id*, *aph(3″)-Ib*, *aadA1*, *aadA2*, *aac(3)-IIa*, *aac(6′)-Ib-cr*, *armA*, *ant(3″)-Ia*), fluoroquinolones (*aac(6′)-Ib-cr*, *qnrB1*), macrolides (*mphA*), phenicols (*floR*, *cmlA1*, *catB3*, *catA1*), rifampicin (*ARR-2*), sulphonamides (*sul1*, *sul2*), trimethoprim (*dfrA14*, *dfrA23*) and tetracyclines (*tetA*). Collectively, these isolates carried ESBL genes, including *bla*_CTX-M-15_ (*n* = 48/49, 98%), as well as *bla*_CTX-M-22_, *bla*_CTX-M-3_; *bla*_CTX-M-216_; *bla*_CTX-M-203_; *bla*_CTX-M-202_; *bla*_CTX-M-176_; *bla*_CTX-M-167_; *bla*_CTX-M-156_; *bla*_CTX-M-103_; *bla*_CTX-M-88_; and *bla*_CTX-M-71_. All the other *bla*_CTX-M_ genes were detected only under AMR profile 24 (*n* = 1/49, 2%). The presence of other ESBL genes, SHV-type (*bla*_SHV-5_), TEM-type (*bla*_TEM-1_ and *bla*_TEM-63_) and OXA-type (*bla*_OXA-1_ and *bla*_OXA-10_) was detected in AMR profile 23, 9–24 and AMR 1–24, respectively. TEM β-lactamases are known to proficiently catalyze the hydrolysis of penicillins and early generation cephalosporins and subsequently confer high-level bacterial resistance against these drugs [8]. The OXA-type β-lactamases confer resistance to ampicillin and cephalothin and are depicted by their high hydrolytic activity against oxacillin and cloxacillin [9]. In addition, plasmid mediated AmpC β-lactamases genes (*bla*_DHA-1_ *bla*_DHA-24;_ *bla*_DHA-7;_ and *bla*_NDM-1_) were identified in AMR profile 24. One isolate under AMR profile 25 harbored one resistance gene (*aac(6′)-Iaa*) conferring resistance only to aminoglycosides. AMR profiles 1–7 were associated with major clusters and are shown below (Table 1). AMR profile 1 (*n* = 13) was the most prevalent AMR profile. Although numerous studies [10–13] have reported correlation between *Salmonella* phenotypic AMR data and genotypic AMR data, data analysis to make correlations to genotypic AMR data was not possible in our study as *Salmonella* isolates reported had incomplete phenotypic AMR data, with many isolates having no phenotypic AMR data.

**Table 1:** AMR gene distribution detected among isolates associated with major clusters

Cluster	Isolates (n)	AMR profile	Beta-lactamases	Aminoglycoside	MLS	Phenicol	Fluoroquinolone	Rifampicin	Sulphonamide	Tetracycline	Trimethoprim
**B, C, D, E**	13	1	*bla*_*CTX-M-15*_*; bla*_OXA-10_*; bla*_OXA-1_	*aac(6′)-Iaa; aph(6)-Id; aph(3″)-Ib; aadA1; aac(3)-IIa; aac(6′)-Ib-cr*	*mph(A)*	*floR; cmlA1; catB3*	*qnrB1; aac(6′)-Ib-cr*	*ARR-2*	*sul2; sul1*	*tet(A)*	*dfrA23; dfrA14*
**D**	1	2	*bla*_CTX-M-15_*; bla*_OXA-10_*; bla*_OXA-1_	*aac(6′)-Iaa; aph(6)-Id; aph(3″)-Ib; aadA1; aac(3)-IIa; aac(3)-IIa; aac(6′)-Ib-cr*	*–*	*cmlA1; floR*	*qnrB1; aac(6′)-Ib-cr*	*ARR-2*	*sul2; sul1*	*tet(A)*	*dfrA23*
**E**	1	3	*bla*_CTX-M-15_*; bla*_OXA-10_*; bla*_OXA-1_	*aac(6′)-Iaa; aph(6)-Id; aph(3″)-Ib; aadA1; aac(3)-IIa; aac(6′)-Ib-crg*	*–*	*floR; cmlA1; catB3*	*qnrB1; aac(6′)-Ib-cr*	*ARR-2*	*sul2; sul1*	*tet(A)*	*dfrA23; dfrA14*
**A**	5	4	*bla*_TEM-1B_*; bla*_OXA-10_*; bla*_CTX-M-15_*; bla*_SCO-1_*; bla*_OXA-1_	*aac(6′)-Iaa; aadA1; aph(6)-Id; aph(3″)-Ib; aac(6′)-Ib-cr*	*–*	*cmlA1; catB3*	*qnrB1; aac(6′)-Ib-cr*	*ARR-2*	*sul1; sul2*	*–*	*dfrA23*
	1	5	*bla*_TEM-1B_*; bla*_OXA-10_*; bla*_CTX-M-15_*; bla*_SCO-1_*; bla*_OXA-1_	*aac(6′)-Iaa; aadA1; aadA1; aph(6)-Id; aph(3″)-Ib; aac(6′)-Ib-cr*	*–*	*cmlA1*	*qnrB1; aac(6′)-Ib-cr*	*ARR-2*	*sul1; sul2*	*–*	*dfrA23*
	1	6	*bla*_TEM-1B_*; bla*_OXA-10_*; bla*_CTX-M-15_*; bla*_SCO-1_*; bla*_OXA-1_	*aac(6′)-Iaa; aph(6)-Id; aph(3″)-Ib; aac(3)-IId; aadA1; aac(6′)-Ib-cr; aadA2*	*mph(A)*	*cmlA1; catB3*	*qnrB1; aac(6′)-Ib-cr*	*ARR-2*	*sul2; sul1*	*–*	*dfrA23; dfrA12*
	1	7	*bla*_TEM-1B_*; bla*_OXA-10_*; bla*_CTX-M-15_*; bla*_SCO-1_*; bla*_OXA-1_	*aac(6′)-Iaa; aph(6)-Id; aph(3″)-Ib; aac(3)-IId; aadA1; aac(6′)-Ib-cr; aadA2*	*mph(A)*	*cmlA1; catB3*	*aac(6′)-Ib-cr*	*ARR-2*	*sul2; sul1*	*–*	*dfrA23; dfrA12*

Seven different AMR profiles (AMR profile 1–7) associated with 5 major clusters (Cluster A-E) were detected among the confirmed 49 *Salmonella* Isangi isolates

*Abbreviations*: *AMR* (antimicrobial resistance), *MLS*, *n* (number of isolates)

### Genotypic relatedness and cluster of the isolates

**Fig. 1:**
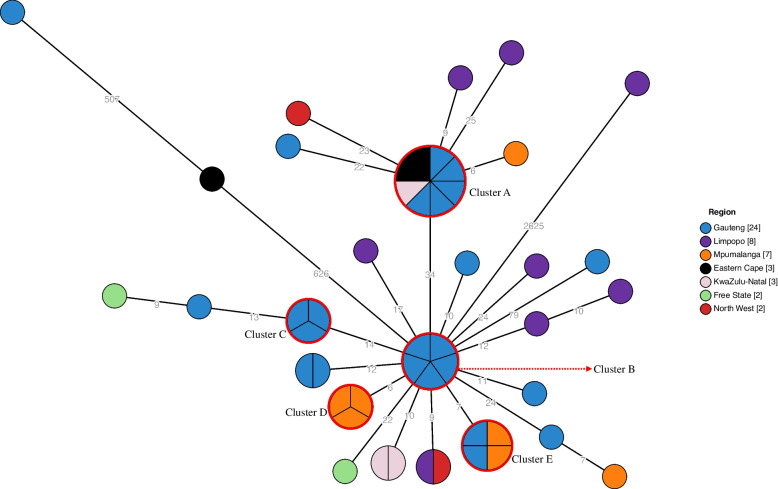
Minimum spanning tree drawn using cgMLST data of *Salmonella* Isangi isolates from South Africa collected over the period 2020–2021. The tree was collapsed to five allele difference threshold. The circular nodes represent isolate(s) having the identical cgMLST profile; the larger the node, the more isolates are reflected. The number of values between adjacent nodes indicates the number of allele differences between nodes (isolates), nodes are colored by region (province)

MLST grouped all MDR and ESBL producing (AMR profile 1–24) *Salmonella* Isangi (48/49, 98%) isolates into one known sequence type, ST335 which was most prevalent. High prevalence of ST335 among South African *Salmonella* Isangi genomes has previously been reported [14]. One non-ESBL producing mono-drug resistant isolate belonging to AMR profile 25 was assigned under ST216 by MLST. The genetic relatedness and cluster analysis of these isolates was investigated using cgMLST, with results depicted in a minimum spanning tree (Fig. 1). Isolates associated with Cluster A (*n* = 8), B (*n* = 5), C (*n* = 3), D (*n* = 3) and E (*n* = 4) were related within five allele differences. The five-allele difference threshold is within the recommended threshold for genetically related *S. enterica* serovars and is indicative of a high probability of epidemiological relatedness [15]. Isolates linked to cluster A had a similar AMR profile (depicted under AMR profile 4–7) and were from the Gauteng (*n* = 5), Eastern Cape (*n* = 2) and KwaZulu-Natal (*n* = 1) Province. Isolates linked to Cluster B (*n* = 5) and C (*n* = 3) had AMR profile 1 and were from the Gauteng Province only. Cluster D isolates (*n* = 3) had an AMR profile of 1 and 2 and were from Mpumalanga Province. Likewise isolates linked to cluster E (*n* = 4) showed AMR profile 1 and 3 and were from Gauteng (*n* = 2) and Mpumalanga (*n* = 2) Province.

## Conclusion

The present study identified *bla*_*CTX-M-15*_ ESBL gene (*n* = 48/49, 98%) and AmpC (*bla*_DHA-1_ and *bla*_NDM-1_) producing strains. ESBLs producing *Salmonella* Isangi in SA have been detected since the early 2000s. The observed high prevalence of *bla*_CTX-M-15_, an ESBL associated gene in MDR *Salmonella* Isangi presents a need for continuous monitoring of this resistance threat in order to mitigate its public health impact. Detecting and determining the degree of ESBLs and AmpC is essential for effective treatment and for prevention and control of multidrug-resistant *Salmonella* serovars. This study therefore provides insight for further studies on antibacterial resistance mechanisms in *Salmonella* Isangi.

### Supplementary Information

**Additional file 1.** Map of South Africa, showing the different provinces of the country. Provinces (regions) are indicated in different colors.

## Data Availability

All sequencing data were uploaded to the public EnteroBase platform (http://enterobase.warwick.ac.uk/species/index/senterica) and so are freely available to access at the EnteroBase platform. In addition, sequencing data are deposited in the European Nucleotide Archive under the project accession numbers PRJEB39546 and PRJEB39988.

## References

[1] Feasey NA, Dougan G, Kingsley RA, Heyderman RS, Gordon MA (2012). Invasive non-typhoidal salmonella disease: an emerging and neglected tropical disease in Africa. Elsevier.

[2] Govinden U, Mocktar C, Moodley P, Sturm AW, Essack SY (2008). Characterization of extended-spectrum β-lactamases in *Salmonella* spp. at a tertiary hospital in Durban, South Africa. Diagn Microbiol Infect Dis..

[3] Kruger T, Szabo D, Keddy KH, Deeley K, Marsh JW, Hujer AM, Bonomo RA, Paterson DL (2004). Infections with nontyphoidal *Salmonella* species producing TEM-63 or a novel TEM enzyme, TEM-131, in South Africa. Am Soc Microbiol..

[4] Tekele SG, Teklu DS, Tullu KD, Birru SK, Legese MH (2020). Extended-spectrum Beta-lactamase and AmpC beta-lactamases producing gram negative bacilli isolated from clinical specimens at international clinical laboratories, Addis Ababa, Ethiopia. PLoS One..

[5] Wadula J, von Gottberg A, Kilner D, de Jong G, Cohen C, Khoosal M, Keddy K, Crewe-Brown H (2006). Nosocomial outbreak of extended-spectrum beta-lactamase-producing *Salmonella* Isangi in pediatric wards. Pediatr Infect Dis J..

[6] Rankin Shelley C, Aceto H, Cassidy J, Holt J, Young S, Love B, Tewari D, Munro Donald S, Benson CE (2002). Molecular characterization of cephalosporin-resistant Salmonella enterica serotype Newport isolates from animals in Pennsylvania. Am Soc Microbiol..

[7] Zhou Z, Alikhan N-F, Sergeant MJ, Luhmann N, Vaz C, Francisco AP, Carriço JA, Achtman M (2018). GrapeTree: visualization of core genomic relationships among 100,000 bacterial pathogens. Cold Spring Harb Lab Press..

[8] Bush K (2002). The impact of β-lactamases on the development of novel antimicrobial agents. Curr Opin Investig Drugs..

[9] Shaikh S, Fatima J, Shakil S, Rizvi SM, Kamal MA (2015). Antibiotic resistance and extended spectrum beta-lactamases: Types, epidemiology and treatment. Saudi J Biol Sci..

[10] Feldgarden M, Brover V, Haft DH, Prasad AB, Slotta DJ, Tolstoy I, Tyson GH, Zhao S, Hsu CH, McDermott PF, Tadesse DA, Morales C, Simmons M, Tillman G, Wasilenko J, Folster JP, Klimke W (2019). Validating the AMR finder tool and resistance gene database by using antimicrobial resistance genotype-phenotype correlations in a collection of isolates. Antimicrobial Agents Chemother..

[11] McDermott PF, Tyson GH, Kabera C, Chen Y, Li C, Folster JP, Ayers SL, Lam C, Tate HP, Zhao S (2016). Whole-genome sequencing for detecting antimicrobial resistance in Nontyphoidal *Salmonella*. Antimicrob Agents Chemother..

[12] Mensah N, Tang Y, Cawthraw S, AbuOun M, Fenner J, Thomson NR, Mather AE, Petrovska-Holmes L (2019). Determining antimicrobial susceptibility in *Salmonella enterica* serovar typhimurium through whole genome sequencing: a comparison against multiple phenotypic susceptibility testing methods. BMC Microbiol..

[13] Neuert S, Nair S, Day MR, Doumith M, Ashton PM, Mellor KC, Jenkins C, Hopkins KL, Woodford N, de Pinna E, Godbole G, Dallman TJ (2018). Prediction of phenotypic antimicrobial resistance profiles from whole genome sequences of non-typhoidal *Salmonella enterica*. Front Microbiol..

[14] Vilela FP, Rodrigues DP, Allard MW, Falcão JP. The rare *Salmonella enterica* serovar Isangi: genomic characterization of the antimicrobial resistance, virulence potential and epidemiology of Brazilian strains in comparison to global isolates. J Med Microbiol. 2023;72(7) 10.1099/jmm.0.001736. 10.1099/jmm.0.001736 37462464

[15] Besser JM, Carleton HA, Trees E, Stroika SG, Hise K, Wise M, Gerner-Smidt P (2019). Interpretation of whole-genome sequencing for enteric disease surveillance and outbreak investigation. Foodborne Pathog Dis..

