# Supplementary figures and images for: Characterization of Salmonella enterica serovar Isangi from South Africa, 2020–2021
Source: BMC Infect Dis. 2023 Nov 13;23:791. doi: 10.1186/s12879-023-08786-9 (PMC10644633; doi:10.1186/s12879-023-08786-9)

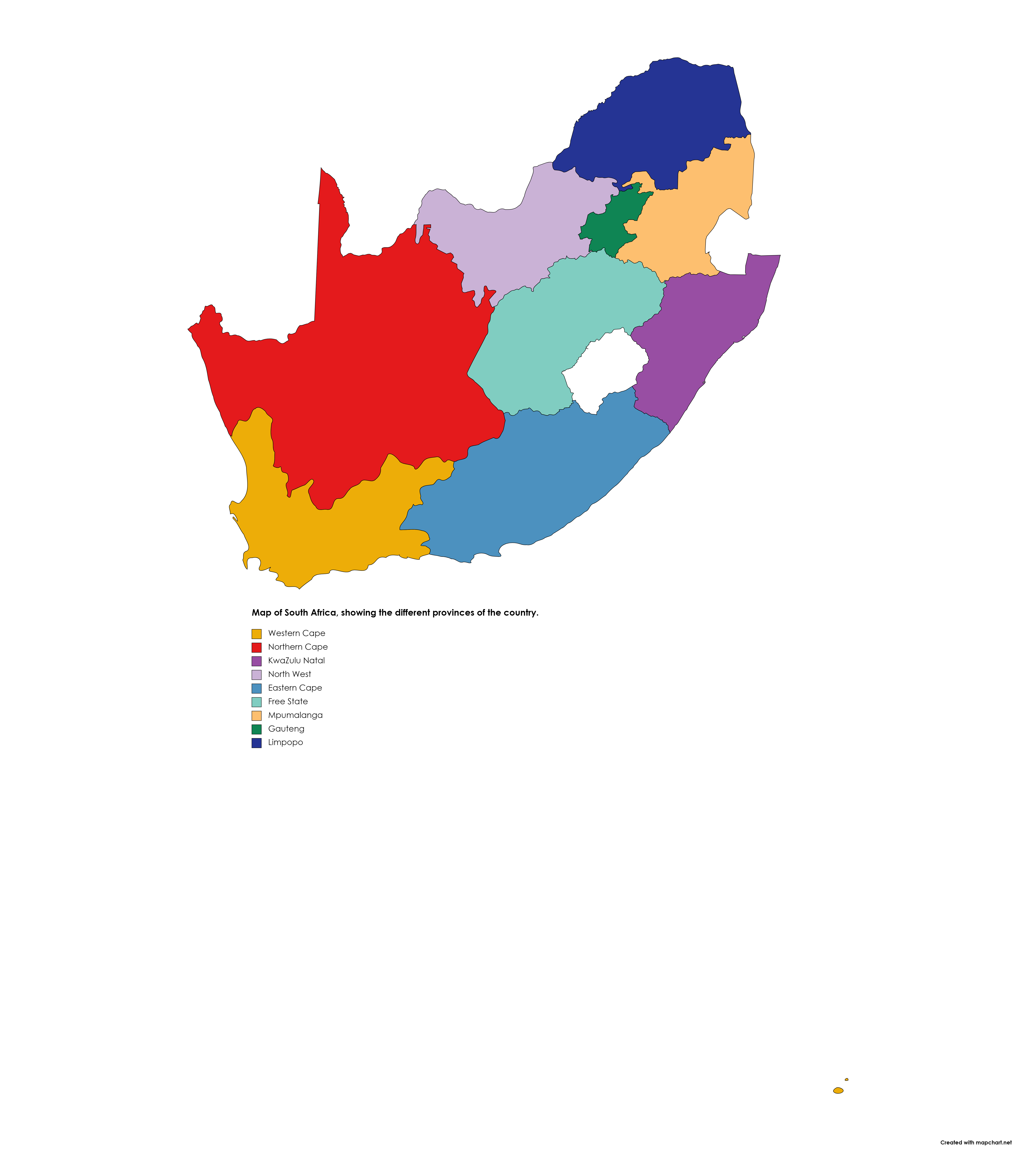

Supplement: Supplementary file 1 — Additional file 1. Map of South Africa, showing the different provinces of the country. Provinces (regions) are indicated in different colors. [file 12879_2023_8786_MOESM1_ESM.png]
